# XH-14, a novel danshen methoxybenzo[b]furan derivative, exhibits anti-inflammatory properties in lipopolysaccharide-treated RAW 264.7 cells

**DOI:** 10.1186/1476-9255-10-1

**Published:** 2013-01-10

**Authors:** Geun-Mook Park, Jong-Gab Jun, Jin-Kyung Kim

**Affiliations:** 1 Department of Biomedical Science, Catholic University of Daegu, 330 Geumrak-Ri, Gyeoungsan-Si, 700-712, South Korea; 2 Department of Chemistry and Institute of Natural Medicine, Hallym University, Chuncheon, 200-702, South Korea

**Keywords:** Inflammation, XH-14, Macrophage, Cytokines, AP-1

## Abstract

**Background:**

XH-14 isolated from *Salvia miltiorrhiza* is a bioactive component and adenosine antagonist. In the present study, we evaluated anti-inflammatory properties of XH-14 in murine macrophages.

**Methods:**

RAW 264.7 murine macrophage cell line was cultured with various concentrations of XH-14 in the absence or presence of lipopolysaccharide (LPS). LPS-induced release and mRNA expression of inflammatory mediators were examined by ELISA and real-time PCR. The modification of signal pathways involved in inflammatory reactions was determined by Western blotting analysis.

**Results:**

XH-14 suppressed the generation of nitric oxide (NO) and prostaglandin E_2_, and the expression of inducible NO synthase and cyclooxygenase-2 induced by LPS. Similarly, XH-14 inhibited the release of pro-inflammatory cytokines induced by LPS in RAW 264.7 cells. The underlying mechanism of XH-14 on anti-inflammatory action was correlated with down-regulation of mitogen-activated protein kinase and activator protein-1 activation.

**Conclusions:**

XH-14 inhibits the production of several inflammatory mediators and so might be useful for the treatment of various inflammatory diseases.

## Background

Inflammation is a basic way in which the body reacts to infection, irritation or other injury, the key feature being redness, warmth, swelling and pain. An inflammatory reaction directs immune system components to the site of injury or infection, and is manifest by increased blood supply and vascular permeability, which allows chemotactic peptides, neutrophils and mononuclear cells to leave the intravascular compartment [1,2]. When an inflammatory reaction is uncontrolled and prolonged, the pathogenesis of chronic diseases such as cancer, arthritis, autoimmune disorder and vascular disease occurs [3]. Because of these reasons, various efforts to regulate inflammatory reactions have been carried, one being to discover dietary phytochemicals capable of suppressing inflammation [4,5].

Various infecting agents, such as bacteria and pro-inflammatory cytokines, can activate macrophages, which are critical immune cells to regulate innate immunity, through certain receptors. The interaction of Toll like receptor (TLR)-4 with the ligand, lipopolysaccharide (LPS), induces an intracellular signaling cascade that activates the mitogen-activated protein kinase (MAPK) family, extracellular signal-related kinase (ERK), p38, C-jun N-terminal kinase (JNK) and adaptor molecules, and ends in the activation of key pro-inflammatory transcription factors such as activator protein (AP)-1 and nuclear factor-kappa B (NF-κB) [6,7]. Eventually, these signaling events lead macrophages to be transcriptionally activated for expressing pro-inflammatory genes including, inducible nitric oxide synthase (iNOS), cyclooxygenase (COX)-2 and various cytokines [7,8].

XH-14 is a bioactive component isolated from *Salvia miltiorrhiza*, also known as Danshen. Danshen is purported to promote circulation and relieve stasis in traditional Chinese medicine [9,10]. XH-14 binds the A1 adenosine receptor with relatively high affinity [10] and XH-14 and their derivatives can inhibit adipocyte differentiation and induction of the adipokines, visfatin and resistin, in 3T3-L1 adipocytes [11]. Although XH-14 has potent biological and physiological functions, the involvement in regulation of inflammatory reactions remains unknown. In this study, we aimed to investigate therapeutic potential of its medicinal benefits against numerous inflammatory diseases by employing *in vitro* inflammatory conditions. In particular, because no study has reported the exact molecular target for the anti-inflammatory effects of XH-14, we focused on finding the inhibitory targets of XH-14.

## Methods

### Chemicals and reagents

XH-14 was synthesized by using a Sonogashira reaction as previously reported [9,12]. LPS derived from *Escherichia coli* and dimethylsulfoxide (DMSO) were obtained from Sigma-Aldrich (St Louis, MO, USA). Dulbecco’s modified Eagle’s medium (DMEM), fetal bovine serum (FBS), penicillin and streptomycin were obtained from Hyclone (Logan, UT, USA). The final concentrations of DMSO never exceeded 0.1%, which did not affect the assay systems. The antibodies (Abs) used were: anti-iNOS rabbit polyclonal, anti-COX-2 monoclonal Ab (mAb), anti-inhibitor of NF-κB (IκBα) mAb, anti-phospho-c-Jun, anti-C-Jun, anti-phospho-c-Fos, anti-c-Fos, anti-phospho-JNK rabbit polyclonal, anti-ERK1/2 rabbit polyclonal, anti-phospho-p38 rabbit polyclonal, anti-p38 rabbit polyclonal, (Cell Signaling Technology, Danvers, MA, USA) and anti-β-actin mAb (Sigma-Aldrich).

### Cell culture and cell viability assay

RAW 264.7 murine macrophages obtained from the Korean Cell Bank (Seoul, Korea) were cultured in DMEM containing 10% FBS, 100 U/ml penicillin and 100 μg/ml streptomycin at 37°C in 5% CO_2_. The effects of XH-14 on cell viability were tested using the CellTiter 96® AQ_ueous_ One Solution Assay of cell proliferation (Promega, Madison, WI, USA). RAW 264.7 cells were plated at a density of 2 × 10^4^ cells in a 96-well flat-bottom plate, and XH-14 were added to each plate at indicated concentrations. After a 24 h incubation period, the number of viable cells was counted according to the manufacturer's instructions. This assay is based on the reduction of a tetrazolium compound, MTS, to formazan, which has an optimum absorption at 490 nm. Thus, the quantity of the product in the cell culture is indicated by the optical density of formazan at 490 nm, which is directly proportional to the number of living cells.

### Measurement of nitrite, prostaglandin E2 (PGE_2_) and cytokines

The amount of nitrite, PGE_2_ interleukin (IL)-1β and IL-6 produced by the mouse macrophages was measured in RAW 264.7 cell culture supernatant. RAW 264.7 cells were plated at a density of 2.5 × 10^5^ cells in a 48-well cell culture plate with 500 μl of culture medium and incubated for 12 h. They were then treated with indicated concentrations of XH-14 plus LPS (100 ng/ml) and incubated for another 24 h. The amount of nitrite and PGE_2_ produced was measured using the Griess reagent system (Promega) and an enzyme-linked immunosorbent assay (ELISA) kit (ENZO Life Sciences, Farmingdale, NY, USA) according to the manufacturer’s instruction, respectively. IL-1β and IL-6 were measured using an ELISA kit (eBioscience, San Diego, CA, USA) according to the manufacturer’s instructions.

### Quantitative real-time reverse-transcription polymerase chain reaction (RT-PCR)

Total RNA was isolated from RAW 264.7 cells and reverse-transcribed into cDNA using TriZol Reagent (Invitrogen, Carlsbad, CA, USA) and CCC TAC CAA GT-3’, R 5’- CAC CCA AAG TGC TTC AGT CA-3’; murine COX-2: F 5’-AAG ACT TGC CAG GCT GAA CT-3’, R 5’-CTT CTG CAG TCC AGG TTC AA -3’; murine IL-1β: F 5’-TTC TCC ACA GCC ACA ATG AG-3’, R 5’-ACG GAC CCC AAA AGA TGA AG-3; murine IL-6: F 5’-CAT CCA GTT GCC TTC TTG GGA-3’, R 5’- CCA GTT TGG TAG CAT CCA TC -3’; GAPDH: F 5’-TCT TGC TCA GTG TCC TTG C-3’, R 5’-CTT TGT CAA GCT CAT TTC CTG G-3’. Real-time PCR assay was carried out with LightCycler (Roche Diagnostics, Germany) using LightCycler FastStart DNA Master SYBR Green I (Roche Diagnostics). Transcripts of glyceraldehyde 3-phosphate dehydrogenase (GAPDH), as a housekeeping gene, were quantified as endogenous RNA of reference to normalize each sample. Relative quantities were estimated by the delta-delta-Ct method. The results were normalized as relative expression in which the average value of the iNOS, COX-2, IL-1β and IL-6 mRNA was divided by the average value of GAPDH mRNA.

### Western blotting analysis

Whole cell extracts, cytoplasmic and nuclear proteins (30 μg protein/lane) were separated by 10% sodium dodecyl sulfate-polyacrylamide gel electrophoresis (SDS-PAGE). The separated proteins were electrophoretically transferred onto nitrocellulose membranes. Immunoreactive bands were detected by incubating the samples with horseradish peroxidase (HRP)-conjugated secondary antibodies and visualized using a WEST-ZOL plus Western Blot Detection System (iNtRON Biotechnology, Seongnam-Si, Korea).

### Statistical analysis

The data are depicted as the mean ± SEM. Student *t*-test was performed using the GraphPad Prism 4.0 software (GraphPad Software, San Diego, CA, USA) and *P* < 0.05 was considered as statistically significant.

## Results

### Effects of XH-14 on the release of NO and PGE_2_

**Figure 1:**
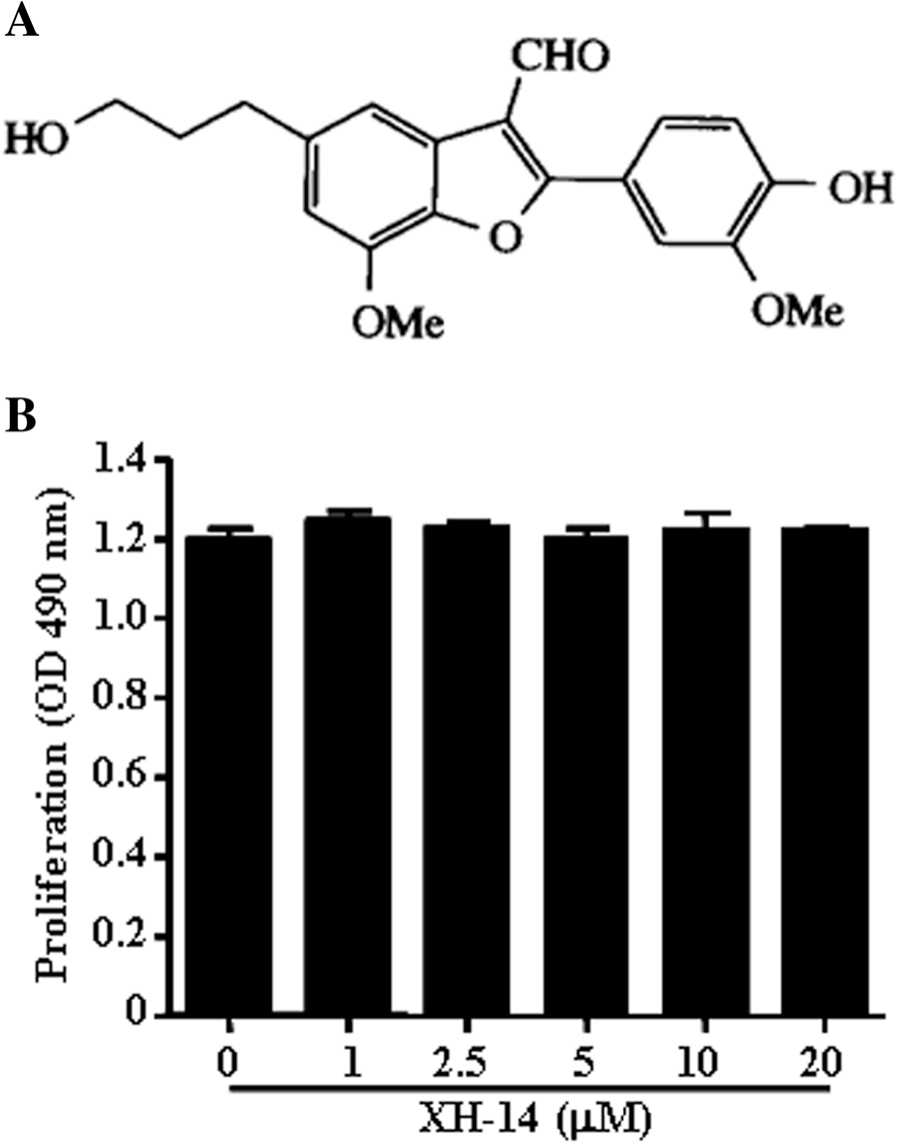
**Effects of XH-14 on murine macrophage viability.** (**A**) Chemical structure of XH-14. (**B**) RAW 264.7 cells were treated with indicated concentrations of XH-14 for 24 h, and proliferation was determined. The results are reported as mean ± SEM of three independent experiments in triplicate.

**Figure 2:**
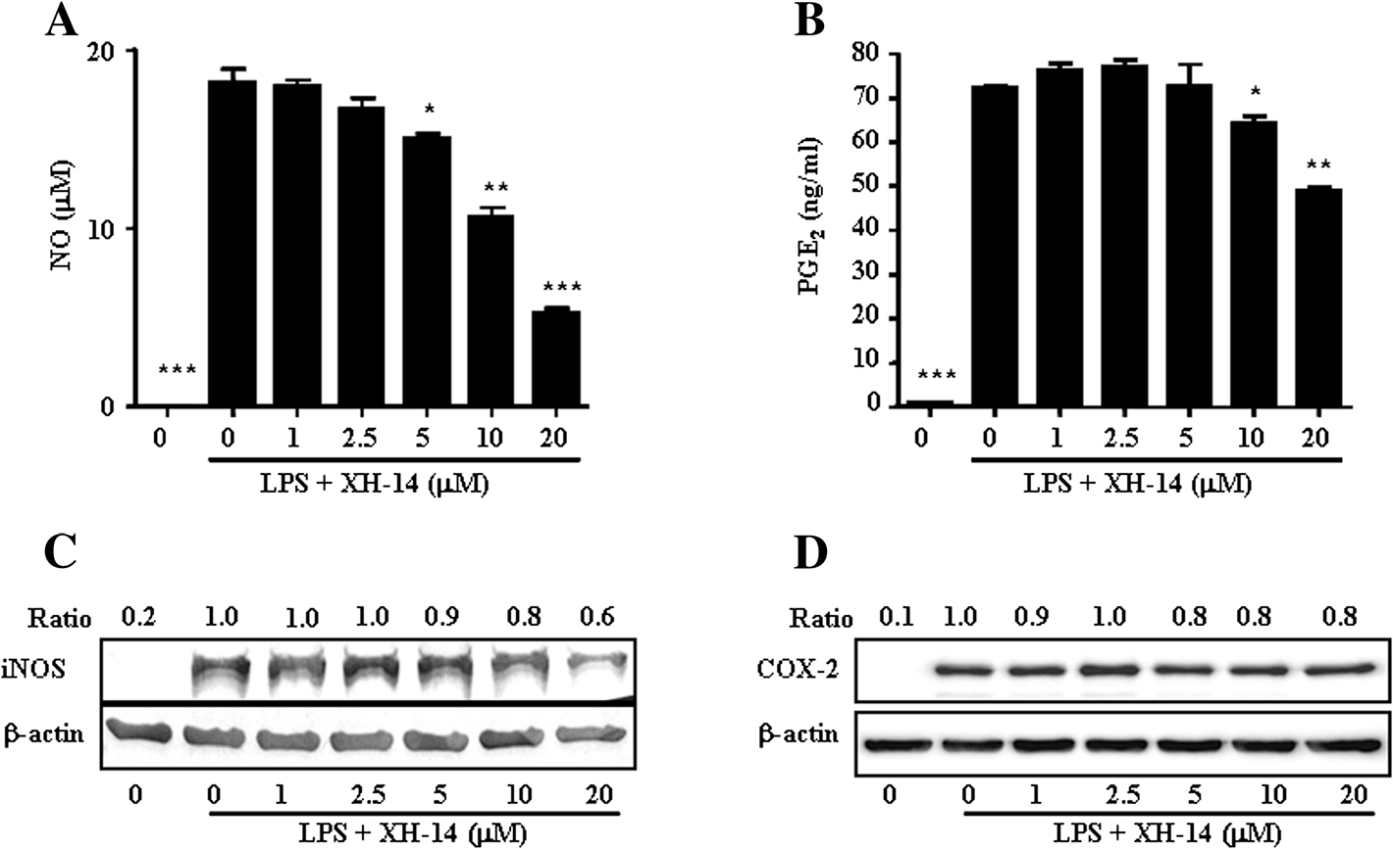
**Effects of XH-14 on LPS-induced NO and PGE**_**2**_**release.** RAW 264.7 cells were treated with 0–20 μM of XH-14 in the presence of 100 ng/ml of LPS or with LPS alone for 24 h, and (**A**) NO and (**B**) PGE_2_ release was determined. The results are reported as mean ± SEM of three independent experiments in triplicate. Statistical significance is based on the difference when compared with LPS-stimulated cells (^*^P < 0.05, ^**^P < 0.01, ^***^P < 0.001). Thirty micrograms of protein obtained from each cell lysate was resolved by 10% SDS-PAGE for (**C**) iNOS and (**D**) COX-2 determination. β-actin expression is shown as a loading control. The bands were quantified using NIH image analysis software and their relative intensity was expressed as fold against the image of the LPS-stimulated RAW 264.7 cells.

To evaluate XH-14-induced anti-inflammatory effects, we used an *in vitro* model with the murine RAW 264.7 macrophage cell line. Since XH-14 showed no cytotoxicity with concentrations up to 20 μM in RAW 264.7 macrophages (Figure 1B), we used up to 20 μM XH-14 for the rest of the experiments. We first sought to determine the effect of XH-14 on the LPS-induced release of the inflammatory mediators, NO and PGE_2_, by RAW 264.7 cells. As shown in Figures 2A and 2B, XH-14 inhibited LPS-induced NO and PGE_2_ production in a dose-dependent manner. The nitrite concentrations in LPS-stimulated cells and in those exposed to 20 μM XH-14 were 18.22 ± 0.74 μM and 5.27 ± 0.22 μM, respectively. The inhibitory effects of XH-14 on PGE_2_ production in LPS-exposed cells were similar to their effects on NO production (Figure 2B). To determine the mechanism by which XH-14 reduces LPS-induced NO and PGE_2_ production, we studied the effect of XH-14 on iNOS and COX-2 protein expression in RAW 264.7 cells using Western blot analysis, since those enzymes catalyze the reaction for NO and PGE_2_, respectively. Consistent with the findings related to NO production, the protein expression of iNOS induced by LPS in RAW 264.7 cells was also reduced by XH-14 treatment (Figure 2C), while having only a marginal effect on COX-2 protein expression.

### XH-14 inhibits the release of pro-inflammatory cytokines in murine macrophages

**Figure 3:**
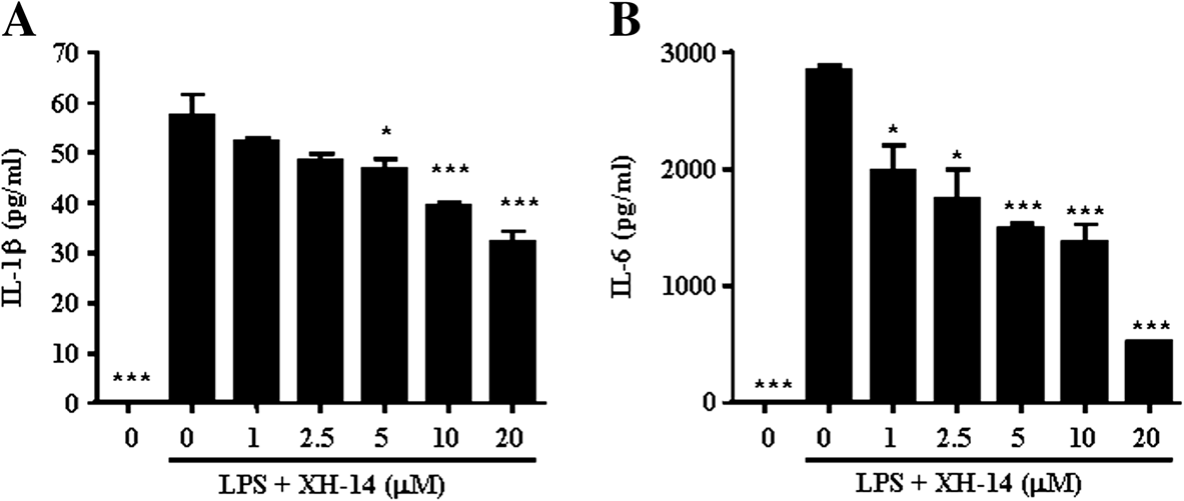
**Effects of XH-14 on LPS-induced inflammatory cytokine production in murine macrophages.** RAW 264.7 cells were treated with 0–20 μM of XH-14 in the presence of 100 ng/ml LPS or with LPS alone for 24 h. The cell culture media were then collected, and the amount of (**Α**) IL-1β and (**B**) IL-6 released was measured. The results are reported as mean ± SEM of three independent experiments in triplicate. Statistical significance is based on the difference when compared with LPS-stimulated cells (^*^P < 0.05, ^***^P < 0.001).

We next examined if XH-14 reduced the release of pro-inflammatory cytokines in LPS-stimulated RAW 264.7 cells. Although the concentrations of IL-1β and IL-6 were not detected in vehicle-treated RAW 264.7 cells, LPS treatment elevated the levels of IL-1β (57.44 ± 4.33 pg/ml) and IL-6 (2842 ± 47.53 pg/ml) in LPS-treated RAW 264.7 cells. XH-14 induced marked suppression of increases induced by LPS in these cytokines (Figure 3). LPS-treated RAW 264.7 cells exposed to XH-14 at concentrations of 1, 5 and 20 μM displayed a dose-dependent inhibited production of IL-1β (10, 16 and 44%, respectively) and IL-6 production (29, 47 and 81%, respectively). These results indicate that XH-14 suppressed various inflammatory mediators including NO and PGE_2,_ as well as pro-inflammatory cytokines, such as IL-1β and IL-6.

### XH-14 inhibits the mRNA expression of iNOS, COX-2, IL-1β and IL-6 in murine macrophages

Since XH-14 suppressed the protein levels of iNOS, COX-2 and pro-inflammatory cytokines in LPS-stimulated RAW 264.7 cells, quantitative real time PCR was used to assess the effects of XH-14 on LPS-induced gene expression of iNOS, COX-2, IL-1β and IL-6 in RAW 264.7 cells. Upon LPS treatment, the mRNA expressions of these four genes were markedly augmented. The concentration response for inhibition of iNOS, COX-2, IL-1β and IL-6 mRNA expressions is shown in Table 1. The results showed that the effect of XH-14 on mRNA expression of iNOS and COX-2 as well as IL-1β and IL-6 was coincidence with the protein expression and secretion of that in supernatants, respectively.

**Table 1:** **Effects of XH-14 on LPS-induced mRNA expression of iNOS, COX-2, IL-1β and IL-6 in RAW 264.7 cells**^**1**^

** mRNA**	**iNOS**	**COX-2**	**IL-1β**	**IL-6**
**(relative expression)**				
vehicle	ND^a^	ND	ND	ND
LPS	1.00 ± 0.00	1.00 ± 0.00	1.00 ± 0.00	1.00 ± 0.00
LPS + 1 μM XH-14	1.14 ± 0.21	0.80 ± 0.23	0.90 ± 0.02	0.74 ± 0.27
LPS + 5 μM XH-14	0.79 ± 0.12^**^	0.69 ± 0.25	0.45 ± 0.06^***^	0.75 ± 0.11^**^
LPS + 20 μM XH-14	0.65 ± 0.03^***^	0.71 ± 0.01^***^	0.37 ± 0.03^***^	0.59 ± 0.05^***^

^1^LPS-stimulated RAW 264.7 cells were cultured with 0–20 μM XH-14 in the presence or absence for 6 h and gene expression was quantified by real-time RT-PCR. The results are reported as mean ± SEM of three independent experiments in triplicate. Statistical significance is based on the difference when compared with LPS-stimulated cells (^**^P < 0.01, ^***^P < 0.001).

^a^ ND; Not detected.

### XH-14-reduced inflammatory mediator release is associated with inhibition of MAPK and AP-1 in murine macrophages

**Figure 4:**
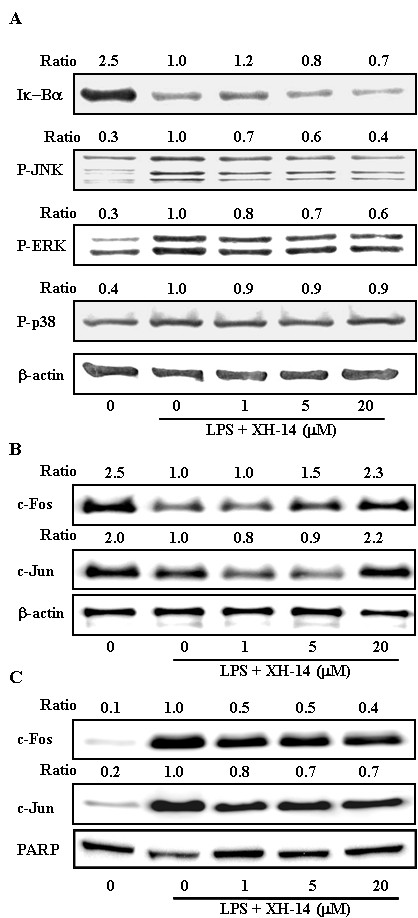
**Effect of XH-14 on LPS-induced MAPK and AP-1 activation.** RAW 264.7 cells were plated in 100 mm-diameter dishes. After 12 h of seeding, cells were treated with different doses of XH-14 for 1 h, followed by stimulation with 100 ng/ml of LPS for 30 min. (**A**) Whole cell extracts, (**B**) cytosolic proteins and (**C**) nuclear proteins were immunoblotted with the indicated Abs. β-actin and poly ADP ribose polymerase were used as a control. The bands were quantified using NIH image analysis software and their relative intensity was expressed as fold-change against the image of the LPS-stimulated RAW 264.7 cells.

Since NF-κB signals regulate the transcription of various genes, including inflammatory mediators, RAW 264.7 macrophages were treated with LPS in the presence or absence of XH-14 and the degradation of Iκ-Bα, an important biochemical event for the nuclear translocation of NF-κB, was determined. As shown in Figure 4A, XH-14 treatment had no effect of the degradation of Iκ-Bα induced by LPS stimulation. In addition to NF-κB, MAPKs pathways are also involved in the regulation of pro-inflammatory mediator expression [13,14]. Treatment with LPS for 30 min resulted in a significant increase in the phosphorylation of JNK, p38 and ERK compared to the vehicle-treated group (Figure 4A). XH-14 markedly prevented LPS-induced increase of JNK and ERK phosphorylation in a concentration-dependent manner, but not phosphorylation of p38 (Figure 4A).

AP-1 is a heterodimeric protein that comprises proteins from various families, including c-Jun and c-Fos [15,16]. Because the expression of AP-1 is under the regulation of the crucial upstream regulatory pathway MAPKs [15-17], we determined the nuclear translocation of c-Jun and c-Fos. As shown in Figure 4B and C, XH-14 inhibited the nuclear translocation of c-Fos and c-Jun induced by LPS stimulation.

## Discussion

XH-14 is one of the natural products isolated from Danshen and is the first-known nitrogen free adenosine receptor ligand. XH-14 is a characteristic member of the neolignan family. Neolignans are widespread in nature. They have a wide variety of chemical structures and exhibit a broad range of biological activities such as anti-cancer [18], anti-proliferative [19], anti-viral [20] and immunosuppressive [21]. Since initial reports that focused on the isolation and synthesis of XH-14, the compound’s biological and physiological activities have remained elusive. Only one study to date has demonstrated a biological function of XH-14, namely the suppressed differentiation of 3T3-L1 adipocytes through the reduction of cellular levels of proliferator activated receptor-γ and CCAAT enhancer binding protein-α [11]. The present study investigated the effect of XH-14 on inflammation.

XH-14 significantly decreased the production of NO, PGE_2_ and pro-inflammatory cytokines in LPS-stimulated murine macrophages. The inhibition of NO was due to the inhibition of iNOS expression at protein and mRNA levels. PGE_2_ was considered one of the strongest inflammatory mediators in inflammatory response. It was transformed from arachidonic acid via the COX-2 catalytic reaction. Although the levels of PGE_2_ and COX-2 mRNA significantly decreased in XH-14 treated RAW 264.7 cells, the expression of COX-2 protein showed anechoic response by XH-14 treatment. The results suggested that the XH-14 might be attributed to its inhibitive effect on PGE_2_ production through blocking COX-2 gene expression as well as activity of COX-2 but not protein expression.

Inflammatory enzymes, iNOS and COX-2, can be used as therapeutic targets to treat various inflammatory diseases. Indeed, non-steroidal anti-inflammatory drugs that suppress COX activities have been used clinically for the treatment of fever, inflammation and pain [22]. In addition, the presence of iNOS in macrophages is significant and, once present, iNOS synthesizes 100–1000 times more NO than the constitutive enzymes and does so for prolonged periods. Since the excessive production of NO and resulting NO-derived metabolites elicit cellular toxicity and tissue damage, which contribute to the pathophysiology of a number of diseases, disorders and conditions, iNOS inhibitors have been investigated for the treatment of iNOS-mediated diseases and conditions including pain, hypotension, inflammation, arthritis and asthma [23]. Based on these facts, XH-14 could be a possible candidate for the treatment of various inflammatory diseases by inhibiting the release of NO and PGE_2_.

The present results provide some preliminary but useful insights into the molecular mechanisms of XH-14. XH-14 did not affect the degradation of Iκ-B, but suppressed the phosphorylation of ERK and JNK as well as nuclear translocation of AP-1, c-fos and c-jun, which were induced by LPS stimulation. To the end of the signaling generated by LPS in macrophages, the activation of transcription factors results in the production of both pro- and anti-inflammatory mediators. The binding of LPS to TLR-4 leads to activation of transcription factor NF-κB and AP-1, which regulate innate immune responses [24]. Activation of NF-κB and AP-1 induces the expression of several inflammatory mediators such as iNOS, COX-2, IL-1β and IL-6, along with many other genes [25,26]. The degradation and phosphorylation of Iκ-B are necessary to release NF-κB from the cytoplasmic NF-κB/IκB complex and allow its subsequent translocation to the cell nucleus. AP-1 is composed of proteins belonging to the Jun and Fos families, and c-jun and c-fos are immediate-early genes [27]. MAPK signaling pathways regulate AP-1 activity by increasing transcription and by the phosphorylation of AP-1 proteins. These results suggest that the inhibition of NO, PGE_2_ and pro-inflammatory cytokines in XH-14 treated RAW 264.7 cells is associated with the down-regulation of AP-1 transcription factors through the inhibition of the MAPK signaling pathway.

## Conclusion

As part of our studies on bioactive constituents from natural products, we previously reported various inhibitors of the release of various inflammatory mediators induced by LPS [28-30]. The present results demonstrate that XH-14 significantly inhibits LPS-induced release of NO, PGE_2_, IL-1β and IL-6, as well as mRNA levels of iNOS, COX-2, IL-1β and IL-6 in RAW 264.7 cells. The same phenomenon was manifested by the inhibitory effects on phosphorylation of ERK and JNK followed by nuclear translocation of AP-1. On the other hand, its inhibitory effects were not shown in Iκ−Bα degradation. A further challenge is to delineate *in vivo* actions of XH-14 to provide a better understanding of the health-promoting effects of a phytochemical that is widely consumed globally.

## Competing interests

The authors declare that they have no competing interests.

## Authors’ contributions

JKK was involved in the design and conduct of the study. GMP performed ELISA, real-time PCR and Western blotting analyses and drafted the manuscript. Preparation of the first draft of the manuscript was done by GMP and the review and approval of the manuscript was performed by JKK. JGJ provided XH-14. All authors read and approved the final manuscript.
